# Optimizing Goal Difficulty in a Digital Weight Loss Intervention: The Ignite Pilot Randomized Trial

**DOI:** 10.1002/osp4.70175

**Published:** 2026-07-19

**Authors:** Michele L. Patel, John A. Gallis, Amanda B. Zeitlin, Phoebe C. Crosthwaite, Annalisa W. Lim, Kayla A. Collins, Marily A. Oppezzo, Lisa G. Rosas

**Affiliations:** ^1^ Stanford Prevention Research Center, Department of Medicine Stanford University School of Medicine Palo Alto California USA; ^2^ Duke Global Health Institute, Duke University Durham North Carolina USA; ^3^ Department of Biostatistics & Bioinformatics Duke University Durham North Carolina USA; ^4^ Department of Pediatrics Stanford University School of Medicine Palo Alto California USA; ^5^ Department of Innovation and Emerging Technology Children's Hospital Los Angeles Los Angeles California USA; ^6^ Stanford University Stanford California USA; ^7^ Johns Hopkins Bloomberg School of Public Health Baltimore Maryland USA; ^8^ Department of Epidemiology & Population Health Stanford University School of Medicine Palo Alto California USA; ^9^ Stanford University School of Medicine Palo Alto California USA

**Keywords:** digital health, goal setting, lifestyle intervention, Multiphase Optimization Strategy, obesity, weight loss

## Abstract

**Background:**

Goal setting is a key component in behavioral weight loss interventions. Goal setting theory emphasizes having harder goals rather than easier goals. However, few studies have experimentally manipulated goal difficulty levels in digital weight loss interventions. Further, when multiple goals are assigned, it is unclear if harder goals are effective or too overwhelming.

**Methods:**

Ignite was a pilot optimization trial guided by the Multiphase Optimization Strategy. A 2^4^ factorial design was used to randomize 32 participants (U.S. adults with overweight or obesity) to either an easier or harder goal for four goal domains: calories, steps, eating windows, and Red Zone Foods (i.e., high‐calorie, low‐nutrition foods). All participants received a 10‐week fully digital weight loss intervention with daily self‐monitoring of goals, weekly lessons, action plans, and feedback. Data were collected via digital tools (daily) and surveys (baseline, 4‐, 10 weeks); feasibility and acceptability were assessed descriptively, while proof of concept was assessed via linear mixed models. Findings were compared to a priori benchmarks.

**Results:**

Participants had a mean (SD) age of 47.7 (13.3) years and BMI of 30.1 (3.8) kg/m^2^ and 47% racial/ethnic minority. Feasibility and acceptability benchmarks were largely met, with high engagement, 94% retention (30/32) at 10 weeks, and 97% recommending the program. For proof of concept, the 3%, but not 5%, weight loss benchmark was met (mean (SD) −3.3 (2.5) kg, or −4.0% (3.6%) at 10 weeks). Participants with a *harder* calorie goal had greater weight loss than those with an *easier* calorie goal (difference: −2.3 kg [95% CI, −4.1, −0.6 kg]). No main effects were observed for other goals.

**Conclusion:**

With high feasibility of study procedures, high engagement, and moderate‐to‐high acceptability, the intervention needs only minor refinements prior to proceeding to a fully powered trial testing the efficacy of easier versus harder goals for weight loss.

**Trial Registration:**

ClinicalTrials.gov NCT05715242. Registered on February 6, 2023

## 1 Introduction

Goal setting is a commonly used behavior change technique for reaching desired health outcomes [1, 2] and is considered vital for behavior change [3, 4, 5]. Goals are defined as the objective of an action representing a desired state, and they usually have a time limit [6]. Goal setting theory, developed by Locke and Latham, emphasizes setting specific (rather than vague) goals and harder (rather than easier) goals, while still ensuring they are attainable [6, 7]. Harder goals have a larger discrepancy between one's current and desired state. An example of a harder, specific goal would be to “walk 2000 more steps every day this week than your average steps” as opposed to an easier, specific goal like “walk 50 more steps every day this week than your average steps” or a vague goal like “walk more steps this week.” Specific and harder goals work through four mechanisms: they direct attention toward relevant activities, produce high effort, promote persistence over time, and encourage the pursuit of new knowledge and skills to attain goals [6, 7, 8].

Goal setting is a core feature of behavioral weight loss interventions, which frequently have multiple concurrent goals (e.g., for caloric intake, physical activity, and weight loss) [9, 10, 11, 12]. With 61%–75% of U.S. adults having obesity [13, 14, 15, 16], there is a pressing need to understand which components in weight loss interventions are driving success, both alone and in combination [17]. By doing so, inactive components can be removed to minimize patient burden and focus efforts on the active components. Digital weight loss interventions serve an important role in delivering these interventions at scale to expand reach and access for the millions of U.S. adults managing this chronic disease [18, 19]. Thus, optimizing digital interventions—including attributes of goal setting (e.g., goal difficulty, specificity, type, number, and tailoring)—would serve to maximize impact while minimizing burden.

Efforts have been made to examine the optimal goal difficulty level. In a meta‐analysis of 155 randomized trials that isolated the effect of goal setting on a range of health behaviors, interventions with hard goals produced greater behavior change than those with easy goals [20]. Observational data also support the use of harder goals, rather than easier goals, when self‐selecting goals, such as greater weight loss for individuals who self‐selected a harder weight loss goal [21, 22, 23, 24, 25, 26]. Several clinical trials have experimentally manipulated goal difficulty levels in health behavior change interventions. Trials that experimentally manipulated caloric intake demonstrated that harder calorie goals produce greater weight loss than do easier goals [27]. However, none of the studies tested digital interventions and most compared very low‐energy diets (i.e., < 800 kcal/day) to low‐energy diets (∼800–1800 kcal/day). In a nutrition‐focused trial testing a 5‐week intervention, participants randomized to a harder nutrition goal (8 servings of low glycemic index foods per day) had comparable dietary outcomes to those with the easier goal (6 servings) [28]. In physical activity research, harder step goals promoted greater increases in step count than easier step goals [29, 30, 31]; however, none were identified in the context of weight loss interventions. In time‐restricted eating interventions, harder (i.e., shorter) daily eating windows produced greater caloric deficits and weight loss than easier (i.e., more lenient) eating windows [32]. Lastly, several trials have compared a “small change approach” (i.e., easier set of goals) to a “large change approach” (i.e., harder set of goals), finding that the large change approach produces greater weight loss [33, 34].

Two key gaps remain. First, the optimal difficulty level of goals in a fully digital weight loss intervention is unknown. Past studies of digital programs have relied on observational data (which support the use of harder weight loss goals [22]); clinical trials are now needed that experimentally manipulate goal difficulty in this context given its potential for widespread scalability. Second, little is known about the optimal difficulty level to target when *multiple* goals are assigned to promote weight loss in a behavioral intervention [35]. For individuals beginning a new health behavior, having multiple challenging goals may be overwhelming, leading to reduced self‐efficacy and effort [8]. Experimental manipulation of goal difficulty across multiple goal domains targeting weight loss would inform goal setting recommendations.

To address these gaps in the goal setting literature for weight loss, a pilot study was designed to test different versions of harder versus easier goals across four goal domains: calorie goal, step goal, eating window goal, and Red Zone Foods goal. These four domains were selected based on their demonstrated efficacy to promote weight loss through mechanisms of caloric restriction and increased energy expenditure [27, 32, 36, 37, 38, 39, 40, 41]. The Multiphase Optimization Strategy (MOST) served as the framework to guide the selection of the optimal difficulty level of goals [42, 43]. Through the Preparation Phase of MOST [44], a pilot trial serves as a small‐scale version of a future fully powered optimization‐RCT, providing an opportunity to examine and refine study procedures and intervention components [45, 46, 47]. MOST balances the desire for maximizing the effectiveness of an intervention with the consideration of real‐world constraints such as patient burden, cost, and scalability. The aims of the Ignite pilot optimization trial were to test the feasibility, acceptability, and proof of concept of a fully digital weight loss intervention—with harder versus easier goals—among adults with overweight or obesity.

## 2 Materials and Methods

### 2.1 Study Design and Overview

**FIGURE 1:**
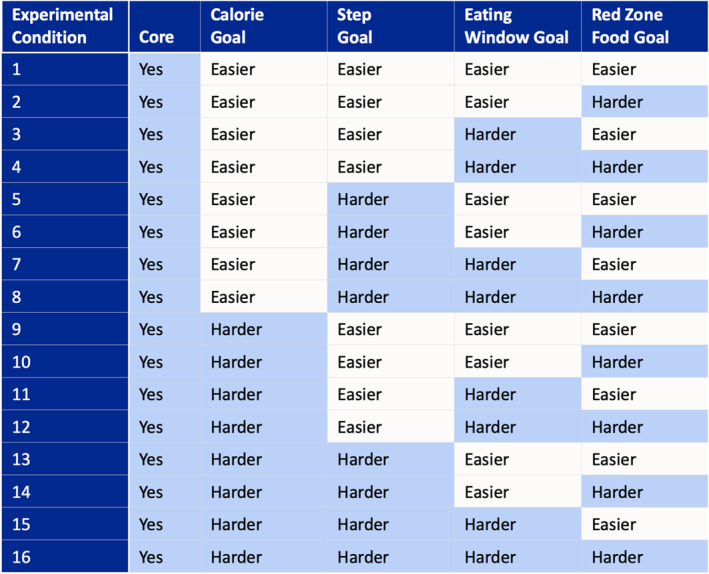
Factorial design (2 × 2 × 2 × 2) testing optimal goal difficulty level across 4 goal domains in the Ignite pilot optimization trial.

Ignite was a pilot optimization trial that used a 2^4^ full factorial design (i.e., 2 × 2 × 2 × 2) to test harder versus easier versions of four goal domains in a 10‐week fully digital weight loss intervention for U.S. adults with overweight or obesity. Each participant was randomly assigned to receive either the harder or easier version of each goal, resulting in 16 experimental conditions (Figure 1). The trial was preregistered at ClinicalTrials.gov (NCT05715242) [48]; the study protocol can be found on that website. No changes were made to the protocol or assessments after the pilot study began. All study procedures and human participant research ethics were approved by the Stanford University Institutional Review Board (protocol #: 68,892; approval date: September 26, 2023). The CONSORT guideline for randomized pilot studies was followed [49].

### 2.2 Participants

Inclusion criteria were the following: adults ages ≥ 18 years, BMI 25.0–45.0 kg/m^2^ (which corresponds to having overweight or obesity [50]), smartphone ownership (Android or iPhone), willingness to install the Fitbit mobile app on their phone, access to a personal email account, English language proficiency, interest in losing weight through behavioral strategies, and living in the United States. Exclusion criteria were the following: concurrent enrollment in another weight management intervention, loss of ≥ 10 pounds in the past 6 months, current use of a weight loss medication, prior or planned bariatric surgery, current or planned pregnancy during the trial period, currently breastfeeding, living with someone else participating in the study, hospitalization for a mental health condition in the past 12 months, inability to engage in moderate forms of physical activity akin to brisk walking (assessed via the Physical Activity Readiness Questionnaire for Everyone [PAR‐Q+] [51]), no medical condition or medication contraindications to weight loss (e.g., end stage renal disease, cancer, schizophrenia, dementia, steroids, anti‐psychotics), if an individual was better suited for a more intensive or different type of intervention based on a health condition (e.g., individuals with a history of an eating disorder or cardiovascular event, uncontrolled hypertension, or uncontrolled diabetes mellitus), and investigator discretion for safety reasons.

### 2.3 Study Procedures

The trial was conducted remotely with participants across the U.S. Assessment visits occurred at baseline, 4 weeks, and 10 weeks.

*Recruitment*. Participants were recruited through several remote channels nationwide and in the San Francisco Bay Area, focusing on (a) ResearchMatch.org, a national registry of volunteers interested in health research, (b) ClinicalTrials.gov, a global registry managed by the NIH of clinical trials, and (c) the Stanford Diabetes Research Center's registry comprised of adults in the San Francisco Bay Area.

*Screening and randomization*. Recruitment materials directed interested individuals to a web‐based eligibility screen (delivered via REDCap, a secure web‐based platform hosted at Stanford University [52]) that described the study and assessed initial eligibility. Eligible individuals then received an email invitation to sign up for a remote baseline visit (held via Zoom videoconference) and to complete an interactive orientation session. The orientation session was self‐directed, lasting 20–25 min, and consisted of video content and interactive activities explaining study expectations, the time commitment, and identification of the pros and cons of trial participation. The orientation session was modeled after the Methods‐Motivational Interviewing Approach [53, 54], designed to increase clinical trial retention by empowering informed choice in trial participation.

At the baseline visit (approximately 60 min), study staff confirmed participant eligibility and interest, described study procedures, obtained electronic informed consent (on REDCap), created a new Fitbit account for each participant, and administered web‐based baseline measures. Once completed, the study staff ordered an e‐scale (Fitbit Aria Air, Google) and an activity tracker (Fitbit Inspire 3, Google) to be sent to participants' homes. Then, participants were sent an email with instructions to sync their devices to the Fitbit app and complete their baseline weight measurements. Study staff were available to troubleshoot if needed. Once both the baseline survey and weight assessment were completed, participants were randomized to 1 of 16 experimental conditions by study staff via an algorithm on REDCap.

Following recommendations for factorial designs [55], permuted block randomization was used with a block size of 16. The allocation sequence was generated by the study's PI in Microsoft Office Excel and stored in REDCap. On the day of randomization, participants received an automated email describing their treatment assignment. Two days following randomization, participants began their first day of the weight loss intervention and received an automated email with their program materials: a goal sheet PDF, a 1‐page “what to expect” PDF, a 6‐min introductory video to Ignite, and a detailed “How to use the Fitbit app” PDF.

*Masking*. Participants were masked to treatment assignment in that no explicit information was provided about the purpose of the trial (experimentally manipulating goal difficulty); rather, the informed consent provided a broader description: “The purpose of the study is to find the best types of goals for promoting weight loss.” Study staff were not masked in treatment assignment due to the need for quality assurance and logistical limitations of conducting a study with few staff members. However, study staff did not have access to the allocation sequence (only the PI did) and all surveys were sent automatically via REDCap.

*Retention strategies*. Participants received compensation for completing assessments: $30 at 4 weeks, $30 at 10 weeks, and an additional $20 for completion of all four dietary recall measures. Automated email reminders were sent to participants the day before their assessment due dates as well as on their due date, and for up to three additional times if not completed. To maximize completion of assessments, study staff collected contact information of a proxy (e.g., a relative or friend) and assessed preferences for contact methods at the baseline visit, and followed up with participants who were due for an assessment via various channels (text message, email, phone call, or contacting proxy after at least 4 attempts).

### 2.4 Intervention

*Overview*. All participants received a 10‐week fully digital weight loss intervention (Table 1). To promote reach and scalability, there was no human counseling provided. Informed by Social Cognitive Theory and Control Theory [56, 57], the intervention included evidence‐based components of goal setting, self‐monitoring, feedback, behavioral lessons, and action planning [2, 4, 9, 10, 58, 59, 60, 61, 62] .These components were designed to promote psychosocial mechanisms of self‐regulation skills and self‐efficacy, which in turn were posited to improve success in each of the four goal domains (reduced caloric intake, eating windows, and Red Zone Foods, along with increased step count) [2, 59, 63, 64], effectively leading to a caloric deficit and weight loss.

**TABLE 1:** Intervention components in the Ignite pilot optimization trial.

Intervention component			Frequency and mode or characteristics
Self‐monitoring			
Dietary intake/calories			Daily via Fitbit app
Steps			Daily via activity tracker (Fitbit Inspire 3)
Eating window			Daily via emailed e‐checklist
Red Zone Foods^a^			Daily via emailed e‐checklist
Body weight			Daily via e‐scale (Fitbit Aria Air)
Goal setting: Experimental components^b^			
*Goal domain*	*Easier*	*Harder*	
Calorie goal: Maximum # of calories to consume each day	Formula + extra 250 kcal/day	Standard formula^c^	Daily; static over time; tailored based on formula to achieve 5% weight loss
Step goal: Minimum # of steps to take each day	40th percentile	60th percentile	Daily; adaptive over time and tailored based on percentile of Fitbit step count in prior week; in 1st week, based on GLTEQ score
Eating window goal: Maximum # of hours to eat within each day	12‐h window	9‐h window	Daily; static over time; untailored
Red Zone Food goal: Maximum # of Red Zone Foods to eat per day	No more than 5 a day	No more than 1 a day	Daily; static over time; untailored
Goal setting: All participants			
Protein goal: Minimum # of grams of protein to consume each day			Daily; static over time; tailored based on 1.6 g per kg of goal body weight
Overall 5% weight loss goal			Over 10 weeks; static over time; tailored based on baseline weight
Weekly weight loss goal of 0.5–2.0 lbs.			Weekly; static over time; tailored based on formula^c^
Tailored feedback			
Feedback on calorie, step, protein, and weight loss goals			Weekly via emailed PDF & daily via Fitbit app
Feedback on eating window and Red Zone Food goals			Weekly via emailed PDF & daily e‐checklist
Skills training			
Lessons: Content on nutrition, physical activity, and behavior change			Weekly via emailed handouts
Action plans: Structured guide to apply week's lesson to own life			Weekly via emailed web‐based form

^a^ Red Zone Foods are foods that are high in calories and low in nutrition (e.g., soda and ice cream).

^b^ Goals were provided on day 1 of the intervention via in an electronic “goal sheet” PDF. Weekly reminders of goals were provided with the feedback PDF.

^c^ Formula for the caloric intake goal is based on the rate of weight loss, which is calculated from baseline‐reported age, sex, height, and weight.

*Experimentally manipulated intervention components*. Participants were randomized to receive either the easier or harder version of each of the following four goal domains:

*Calorie goal (easier* vs. *harder*). At baseline, a tailored goal for caloric intake was calculated based on an individual's baseline height, weight, sex, and age in order to achieve 5% weight loss by 10 weeks [65]. For safety, a minimum goal of 1200 Calories (kcal)/day for women and 1500 kcal/day for men was set based on national guidelines [50]. Participants were randomized to receive either an *easier* calorie goal (i.e., the tailored calorie goal plus 250 extra calories in their daily budget) or a *harder* calorie goal (i.e., the tailored calorie goal as is).

*Step goal (easier* vs. *harder).* At baseline, a tailored step goal was computed based on the leisure score index from the Godin Leisure‐Time Exercise Questionnaire [66, 67]. Participants with scores of 0–13 (interpreted as “Insufficiently Active”) were assigned an initial baseline goal of 5000 steps per day, scores of 14–23 (“Moderately Active”) were assigned 7000 steps per day, and scores ≥ 24 (“Active”) were assigned 10,000 steps per day. The minimum goal of 5000 steps per day was selected based on an average step count of 5000 steps among adults nationwide and globally [68, 69], and lower step counts, on average, among adults with overweight or obesity [70]. Participants were randomized to receive either an *easier* step goal (i.e., the tailored baseline step goal as is) or a *harder* step goal (i.e., the tailored baseline step goal plus 1500 additional steps per day). Beginning in week 2, this step goal then became adaptive such that it varied based on the prior week's step count using an empirically tested algorithm [71, 72]. Specifically, participants received a daily step goal of either the 40th percentile‐rank (if easier version) or 60th percentile‐rank (if harder version) of the prior week's daily step count, rounded up to the nearest 500 steps. For example, a week with daily steps of 3000, 3280, 4550, 5200, 6300, 9010, and 11,500 (ranked lowest to highest) would result in a step goal of 4810 steps (which would be rounded up to 5000 steps) for the easier version and 5860 (which would be rounded up to 6000 steps) for the harder version. The maximum goal that could be assigned was 15,000 steps per day.

*Eating window goal (easier* vs. *harder).* Limiting eating windows is the crux of time restricted eating, and it aids in weight loss due to reduced caloric intake of 200–550 kcal per day, such as from eliminating late‐night snacking [32, 36]. U.S. adults have an average eating window of 14–15 h per day [73]. Participants randomized to the easier version of this goal were instructed to eat within a 12‐h window every day, while those randomized to the harder version were instructed to eat within a 9‐h window every day. At baseline, participants self‐selected their preferred eating window (e.g., 9 a.m. to 9 p.m. for someone in the easier version, or noon to 9 p.m. for someone in the harder version); instructions were provided on maintaining this eating window for the duration of the intervention. Zero‐calorie beverages (e.g., water, tea, coffee, diet soda) were permitted outside of the daily eating window.

*Red Zone Food goal (easier* vs. *harder).* Red Zone Foods are foods high in calories and low in nutrition, such as soda, white bread, and fried chicken. These have been referred to in other works as “indulgences,” “discretionary foods,” or “energy‐dense, nutrient‐poor foods.” [74, 75] Based on the Traffic Light Diet [37, 38] limiting Red Zone Foods may provide a simplified strategy for reducing caloric intake to aid in weight loss [39, 76]. Participants randomized to the easier version of this goal were instructed to eat no more than 5 Red Zone Foods per day, while those randomized to the harder version were instructed to eat no more than 1 Red Zone Food per day.

*Core components*. All participants received a core intervention consisting of the following components: a 5% weight loss goal by the end of the 10‐week intervention with a corresponding weekly weight loss goal of 0.5–2.0 lbs. per week (i.e., 0.23–0.91 kg per week) [77]; a daily protein goal (1.6 g per kg of goal body weight), an amount recommended when in a caloric deficit or period of weight loss [78, 79]; weekly skills training and feedback; and daily self‐monitoring of four behaviors (1) body weight via an e‐scale, (2) dietary intake, including all foods and drinks consumed and their corresponding caloric intake and protein intake via the Fitbit mobile app (now called the Google Health app), (3) steps via the wrist‐worn Fitbit Inspire 3 activity tracker, and (4) eating window via a web‐based e‐checklist sent each morning that prompts eating start and stop times from the prior day (automatically sent via REDCap). Participants were also instructed to self‐monitor Red Zone Foods via the same e‐checklist (REDCap) daily in weeks 1 and 10 and once per week in weeks 2 through 9. Prior to day 1 of the intervention, the study staff set up the Fitbit app with participants' tailored goals for weight loss, step count, caloric intake, and protein intake.

Feedback was provided on both a daily and weekly basis. For behaviors tracked via or synced with the Fitbit app (weight, steps, calories, protein), progress could be viewed in real‐time in graphical or numerical format. For behaviors tracked via the daily e‐checklist (eating windows, Red Zone Foods), immediate, automated feedback was provided upon submitting the e‐checklist as to whether goals were met. In addition, each week, participants received an email with a “progress report” in PDF format of tailored feedback on their assigned goals. Feedback included the following: (a) total number of days in prior week of self‐monitoring diet, steps, and food (each reported separately), (b) total weight change since starting the intervention and distance from weight loss goal, (c) change in weight and Red Zone Food intake the prior week compared to 2 weeks prior, (d) mean of prior week's behaviors (daily caloric intake, daily step count, daily eating window, daily protein intake), and (e) whether each of these goals were met along with the total number of goals met that week.

On a separate day each week, participants received an email with skills training materials that included structured behavioral lessons in PDF on nutrition and physical activity topics (e.g., reading nutrition labels, navigating the grocery store, reducing added sugar, and incorporating new exercises). Corresponding action plans in a web‐based form (Qualtrics) were also provided. The action plans pertained to the weekly lesson and incorporated reflection of current behaviors, identification of areas for change and strategies to do so, motivational interviewing techniques [80, 81], problem‐solving strategies [82], and identification of people who could promote social support. Action plans have been shown to promote behavior change [59, 83, 84] potentially through increased self‐regulation or habit formation. Automated reminders via email were sent to reinforce the completion of action plans. These skills training materials were adapted from the investigator team's recent trials [39, 85] as well as from gold standard weight loss curricula [58].

REDCap was used to automate the delivery of all intervention materials. Staff facilitated this process by downloading data each week from Fitbit using a third‐party software platform called Fitabase (e.g., calorie data) or from REDCap (e.g., eating window data), then pasting these data into an Excel spreadsheet, which had an algorithm to automatically generate personalized feedback; staff then used Microsoft Mail Merge to create PDFs of each progress report, and finally uploaded them to REDCap.

### 2.5 Measures

The primary outcomes of this pilot study were related to the feasibility and acceptability of the various goals and their difficulty levels. Study assessments were completed at baseline, 4 weeks, and 10 weeks—all conducted remotely. To characterize the sample, baseline measures via a web‐based survey were collected, assessing sociodemographic (e.g., sex, age), clinical (e.g., diabetes diagnosis, smoking history), and behavioral (e.g., history of self‐monitoring [86]) domains, as well as the orally‐administered Newest Vital Sign to assess health literacy [87]. The assigned baseline goals are also reported, since many were tailored rather than standardized.

*Manipulation check for goal difficulty*. The perceived goal difficulty was assessed for each of the four experimentally manipulated goals (calories, steps, eating window, Red Zone Foods) as well as of the protein goal and weekly weight loss goal. Response options were on a four‐point Likert scale ranging from *very hard*, *somewhat hard*, *somewhat easy*, to *very easy*, and also had an *not applicable* option. No pre‐established benchmarks were set for the manipulation check.

*Feasibility measures*. Enrollment rate was defined as the percentage of individuals who enrolled in the study out of those who were eligible after completing the online eligibility screen. The recruitment rate was operationalized as the number of participants who consented divided by the number of weeks recruitment occurred (i.e., the dates from the first to last informed consent).

Engagement in the 10‐week intervention was assessed for the components of self‐monitoring, lessons, and action plans. Engagement in self‐monitoring was calculated as the total number of days of self‐monitoring out of total days self‐monitoring was prescribed (i.e., 70 days for all domains except Red Zone Foods, which was 22 days) × 100. It was measured objectively using data derived from the digital tools and was reported as the percent of days in the intervention that participants self‐monitored dietary intake/calories, steps, eating window, Red Zone Foods, and body weight (reported separately for each domain). A software data management platform (Fitabase; Small Steps Lab LLC) retrieved data from the Fitbit app, while REDCap collected and stored data from the e‐checklist. For self‐monitoring of diet, only days with ≥ 800 kcal recorded were considered valid [88]. For self‐monitoring of steps, only days with ≥ 1000 steps were considered valid [89]. Engagement in lessons was defined as the percentage of the 9 lessons reviewed, as self‐reported via a survey at 10 weeks; participants who did not complete this survey were assumed to have read 0% of lessons. Engagement in action plans was defined as the percentage of the 9 action plans completed, which was objectively assessed via a Qualtrics survey. For engagement in feedback, the 3‐month survey asked participants to report their frequency of reading their weekly progress reports, with response options of *weekly*, *less than 1 time per week*, *less than 1 time per month*, or *never read my progress reports*.

Goal attainment in the 10‐week intervention was calculated as the percentage of days a goal was met (for goals pertaining to calories, steps, eating window, Red Zone Foods, and protein) or as the percentage of weeks the weight loss goal was met. The denominator used for this calculation was 70 days for the calorie and protein goals, 69 days for the eating window goal since day 1 was not included because it asked about their eating window before the intervention began, 13 days for the Red Zone Food goal since self‐monitoring of this domain only occurred daily in weeks 1 and 10 (and day 1 was excluded for the same reason as described above), 63 days for the step goal to standardize how many days participants had with each new step goal given that day 1 of the intervention could start on any day of the week, and 10 weeks for the weekly weight loss goal. For the eating window goal, it was considered as met if the participant ate within their maximum allotted hours per day (i.e., 9 or 12 h); the actual start and stop times did not factor into this calculation. Days without any self‐monitoring or without valid data were considered not met.

Retention was defined as the percent of participants who submitted a weight entry via either the synced e‐scale or weight survey at a given time point, while survey completion was defined as those who submitted the web‐based survey at a given time point.

Participants were encouraged to report any adverse events or discomforts that arose during the trial period to the study team and were provided with the team's contact information on multiple occasions. If reported, staff would complete a templated data entry form on REDCap to document adverse event information.

*Acceptability measures*. In the 10‐week survey, a series of questions assessed the acceptability of the intervention, including “Would you recommend the Ignite weight loss program to a friend who is trying to lose weight?” with *yes* and *no* response options and “How helpful did you find each of the Ignite intervention components?” (13 components assessed), with response options of *not at all helpful*, *somewhat helpful*, *moderately helpful*, *very helpful*, and *extremely helpful*.

*Proof of concept measures*. The planned *primary* outcome for a future, fully powered optimization trial is weight change. Body weight was measured using study‐provided e‐scales (Fitbit Aria Air) that transmit data to the Fitbit app; there was high concordance between weight measurements from e‐scales in participants' homes and those from clinical settings [90, 91]. For each weight assessment (baseline, 4 weeks, 10 weeks), participants were provided with best practices to submit a weight entry [92]: place the scale on a hard surface, weigh in the morning without clothing or shoes, before eating/drinking, and after voiding, and repeat for 3 total measurements per assessment. Participants were instructed to sync their scale to the app immediately during the weight assessment and, as a preventive measure in case of syncing issues, to also fill out a web‐based survey (REDCap) with these weight values. Based on prior research on fully digital weight loss interventions [19, 93, 94, 95, 96, 97, 98, 99, 100, 101], benchmarks were set for the proportion of participants achieving weight loss of ≥ 3% and ≥ 5%, which have been considered clinically meaningful given their associations with improved cardiometabolic health [50, 102]. Analyzes assumed that individuals who did not submit a weight entry at 10 weeks did not achieve either of the clinically significant benchmarks.

Health behaviors were also assessed using measures to be tested in a subsequent, fully powered optimization trial. Caloric intake, Red Zone Food intake, and protein intake were assessed via the Automated Self‐Administered 24‐Hour (ASA24) Dietary Assessment Tool (version 2022) [103]. At baseline and 10 weeks, participants were instructed to complete one weekday recall and one weekend day recall, with the average of them being reported. Recalls with values < 600 kcal or > 4400 kcal for women and < 650 kcal or > 5700 kcal for men were excluded from analyses [104]. Physical activity was measured in two ways: via self‐report using the GLTEQ self‐report measure to assess change from baseline to 4 and 10 weeks as well as objectively via the Fitbit Inspire 3 activity tracker to evaluate step count during the intervention period, which has high validity [105]. The GLTEQ assesses the past week's frequency of strenuous (e.g., running), moderate (e.g., fast walking), or mild (e.g., easy walking) activities during one's free time; it computes a leisure score index using the formula (strenuous × 9) + (moderate × 5) + (mild × 3), as well as a MVPA score index that excludes mild activities [66, 67]. Higher values indicate greater activity levels, with scores on the MVPA score index of ≥ 24 units interpreted as *active*, and scores < 24 interpreted as *insufficiently active* [106]. Adherence to the U.S. physical activity guidelines was assessed via the one‐item Stanford Leisure‐Time Activity Categorical Item (L‐CAT) [107]; it included 6 response options, with three describing insufficiently active levels of physical activity and the other three describing sufficiently active levels, according to the guidelines. For steps, change in mean step count was computed from week 1 to week 10 of the intervention, along with average daily step count during the intervention.

In exploratory analyses, change in daily eating window duration from baseline to 10 weeks was assessed via survey items asking about the start and stop eating times of the prior day. Change in daily Red Zone Food intake from baseline to 10 weeks was assessed by uploading deidentified ASA24 dietary intake data into ChatGPT Plus along with the Red Zone Food checklist, which was then prompted to flag Red Zone Foods consumed and calculate the daily intake per recall. The mean of the weekday and weekend recalls were computed per participant per time point. To check the accuracy of these AI‐generated intake values, a study staff member manually flagged all Red Zone Foods, and the investigator team trained ChatGPT Plus to include any terms that it did not flag. Lastly, changes in daily protein intake from baseline to 10 weeks were assessed using ASA24 dietary recalls.

### 2.6 Sample Size and Statistical Power

Aligning with recommendations for conducting pilot studies [45, 46, 47], no power analysis was conducted; instead, the pilot study focused on the feasibility and acceptability of the intervention and study procedures. The sample size (*n* = 32) was selected to have the same number of participants per condition and allowed us to meet budget and time restrictions.

### 2.7 Statistical Analysis

Descriptive data were used to assess feasibility and acceptability outcomes, and were compared to a priori benchmarks. Medians and interquartile ranges were reported for non‐normally distributed data. In exploratory analyses, proof of concept was evaluated of the intervention on weight loss, which is the investigator team's planned primary outcome of a subsequent, fully powered optimization trial. To do so, the main effects of each of the four goal‐setting components that were experimentally manipulated (comparing the harder vs. easier versions) were assessed, along with their interactions, using linear mixed models with effect coding (−1 for easier vs. +1 for harder version of a goal) [43], in an intent‐to‐treat approach. The linear mixed model provides valid inference under the missing at random assumption, conditional on the covariates included in the model [108].

To interpret results from factorial trials, conditions should not be compared to each other (1 vs. 2 vs. 3, etc.), but rather *combinations* of conditions are compared to test main effects and interactions of each factor‐level [109] (i.e., the harder vs. easier versions of the four goal domains). e.g., the main effect of calorie goal difficulty on weight change is calculated by comparing the mean weight change for the participants who received the easier version of it (i.e., experimental conditions 1–8 in Figure 1) versus the mean of those who received the harder version of it (i.e., conditions 9–16). Because each analysis in a 2^k^ factorial trial (*k* = number of factors being tested) uses the entire sample, it is considered an efficient and economical study design [43]. Analyses were conducted using SAS 9.4 (SAS Institute, Cary, NC).

## 3 Results

### 3.1 Trial Overview

**FIGURE 2:**
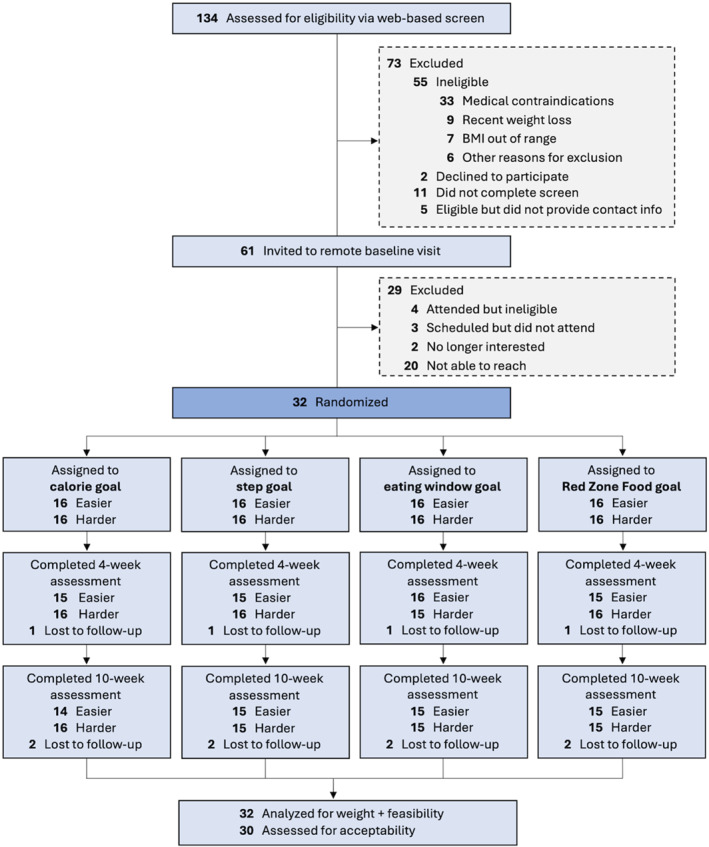
CONSORT flow diagram. Weight change is the planned primary outcome of the subsequent fully‐powered trial; thus, retention at the 4‐ and 10‐week assessments refers to weight entries being completed.

Enrollment in the Ignite pilot study lasted 7 weeks, beginning on December 5, 2023 and ending on January 23, 2024 once the target of 32 participants was reached. Data collection ended on April 14, 2024. Among 134 individuals who took the eligibility screen, 46% (*n* = 61) met eligibility criteria and were invited to the remote baseline visit; of those, 52% (*n* = 32) were enrolled and randomized in the trial. As depicted in the CONSORT flow diagram, half of the participants were randomized to receive the harder version of each goal domain and half the easier version (Figure 2). The recruitment rate was a mean of 4.6 participants enrolled per week. No participants formally withdrew or were withdrawn from the trial after randomization. Trial retention was 97% (31/32) at 4 weeks and 94% (30/32) at 10 weeks. Reasons for missed assessments were unknown.

### 3.2 Participant Characteristics and Goals

Baseline characteristics included the following: mean (SD) BMI of 30.3 (3.6) kg/m^2^ and age of 47.7 (13.3) years; 75% female and 25% male; 44% having obesity and 56% having overweight; and 47% identifying as a racial/ethnic minority (Table 2; additional details in Supporting Information S1: Table S1). Participants were recruited from 13 U.S. states.

**TABLE 2:** Baseline characteristics of Ignite participants.

Characteristic	Total (*N* = 32)
Clinical characteristics	
Weight, kg, mean (SD)	85.7 (11.9)
BMI, kg/m^2^, mean (SD)	30.3 (3.6)
BMI category, *N* (%)	
Has overweight, 25–29.9 kg/m^2^	18 (56.3)
Has obesity, 30–45.0 kg/m^2^	14 (43.8)
Limited health literacy (NVS), *N* (%)	4 (12.5)
Prediabetes or type 2 diabetes, *N* (%)	9 (28.1)
Hypertension, *N* (%)	6 (18.8)
Sociodemographic characteristics	
Age, y, mean (SD)	47.7 (13.3)
Sex assigned at birth, *N* (%)	
Male	8 (25.0)
Female	24 (75.0)
Married or living with partner, *N* (%)	19 (59.4)
College graduate (4 years) or more, *N* (%)	23 (71.9)
Employed full‐time, *N* (%)	20 (62.5)
Race/ethnicity, *N* (%)^a^	
Hispanic (any race)	5 (15.6)
Non‐Hispanic White	17 (53.1)
Non‐Hispanic Black	5 (15.6)
Non‐Hispanic Asian/Native Hawaiian/Pacific Islander	3 (9.4)
Non‐Hispanic multi‐racial	2 (6.3)
Behavioral characteristics	
Self‐monitoring history in the past month, *N* (%)	
Never tracked diet	20 (62.5)
Never tracked steps	11 (34.4)
Never tracked weight	5 (15.6)
Caloric intake, kcal/day (ASA24), mean (SD)	1950.9 (673.1)
Step count, steps/day^b^ (Fitbit Inspire 3), mean (SD)	8787 (3773)
Physical activity (GLTEQ)	
MVPA score index, mean (SD)	25.0 (22.8)
Leisure score index, mean (SD)	35.2 (28.9)
Active, *N* (%)	19 (59.4)
Moderately active, *N* (%)	6 (18.8)
Insufficiently active, *N* (%)	7 (21.9)
Eating window	
Eating window duration, hours/day, mean (SD)	11.7 (2.9)
Eating start time, hh: mm, mean (SD)	09:06 a.m. (02:10)
Eating end time, hh: mm, mean (SD)	08:50 p.m. (02:25)
Red Zone Foods intake, count/day (ASA24), mean (SD)	5.9 (2.4)
Protein intake, grams/day (ASA24), mean (SD)	78.1 (28.6)

Abbreviations: ASA24, Automated Self‐Administered 24‐Hour (ASA24) Dietary Assessment Tool; BMI, body mass index; GLTEQ, Godin Leisure‐Time Exercise Questionnaire; hh:mm, hours (2 digits) and minutes (2 digits) of time‐of‐day using the 12‐h clock; kcal, kilocalorie; MVPA, moderate‐to‐vigorous physical activity; NVS, Newest Vital Sign health literacy measure.

^a^ No participants who identified as Non‐Hispanic selected “American Indian or Alaska Native”.

^b^ Reported data refer to the mean step count in week 1 since participants did not begin wearing the Fitbit tracker until the start of the intervention.

The assigned goals during the intervention were as follows: mean (SD) calorie goal of 1371 (258) kcal/day for harder and 1581 (174) kcal/day for easier, step goal in week 1 of 10,000 (2098) steps/day for harder and 8500 (2098) steps/day for easier (N.B., this goal adapted each week), eating window goal of 9 (0) hours per day for harder and 12 (0) hours per day for easier, Red Zone Food goal of 1 (0) per day for harder and 5 (0) per day for easier, protein goal of 127 (18) g per day across all participants, and weekly weight loss goal of 1.0 lbs. for 23 (72%) participants and 1.5 lbs. for 9 (28%) of participants. The mean self‐selected start and stop times for eating windows were 10:28 a.m. to 07:28 p.m. for the harder goal and 08:23 a.m. to 08:23 p.m. for the easier goal.

### 3.3 Manipulation Check for Goal Difficulty

**FIGURE 3:**
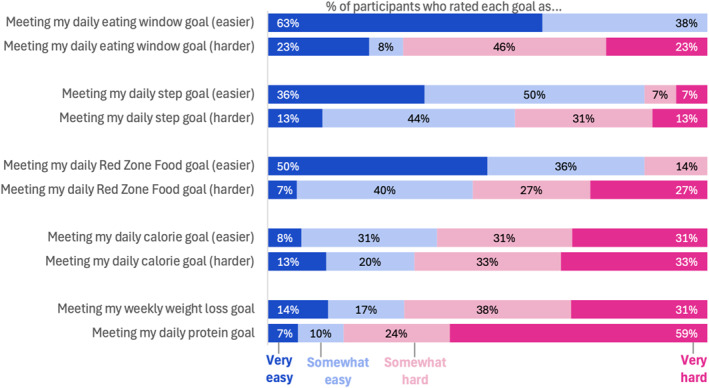
Perceived goal difficulty. Some goal domains may not add up to 100% exactly due to rounding. Four goals had their difficulty level experimentally manipulated to an easier versus harder version: calorie goal, step goal, eating window goal, and Red Zone Food goal. The weekly weight loss goal and protein goal were not experimentally manipulated.

The percentage of participants who perceived each goal as *somewhat* or *very* hard was higher, descriptively, for the harder versus easier version of all domains: calorie goal (66% vs. 62%), step goal (44% vs. 14%), eating window goal (69% vs. 0%), and Red Zone Food goal (54% vs. 14%). For the goals that were not experimentally manipulated, 69% of participants perceived the weekly weight loss goal as *somewhat* or *very* hard as did 83% for the protein goal (Figure 3).

### 3.4 Feasibility Outcomes

**FIGURE 4:**
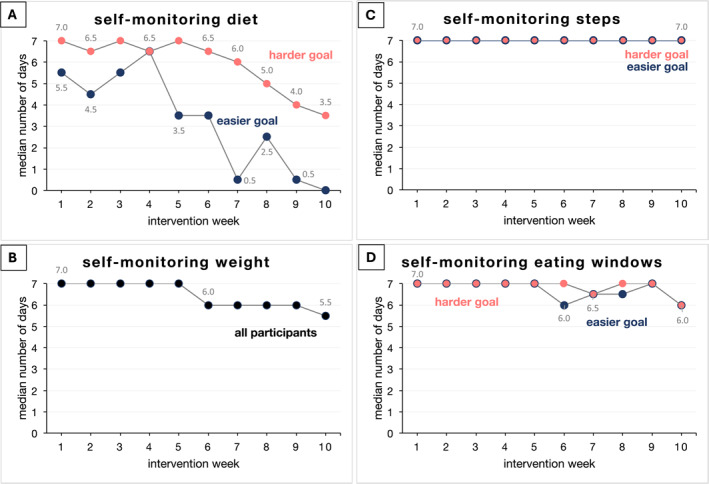
Self‐monitoring engagement over the 10‐week Ignite intervention by goal difficulty. Engagement was operationalized as the median number of days per week of self‐monitoring for a given domain (panel (A) dietary intake and corresponding calories, panel (B) weight, panel (C) steps, panel (D) eating windows). Participants were instructed to self‐monitor these domains daily. Goal difficulty levels (light pink for harder, dark blue for easier) are shown for all self‐monitoring domains except self‐monitoring weight (panel B), which was not experimentally manipulated.

Among all participants, feasibility benchmarks were met for the recruitment metric, 7 of 7 (100%) intervention engagement metrics, 1 of 5 (20%) goal attainment metrics, and 5 of 5 (100%) assessment completion metrics (Table 3). Engagement in self‐monitoring ranged from a low of a median 76% [IQR: 38%–93%] of days for tracking dietary intake to 99% [IQR: 95%–100%] of days for tracking steps. Descriptively, engagement in self‐monitoring remained high over the 10‐week intervention for all goal domains that had daily self‐monitoring prescriptions, except self‐monitoring dietary intake, which declined over time and had a more pronounced drop with the easier calorie goal than the harder calorie goal (Figure 4). One participant never self‐monitored any domain, and one participant self‐monitored every domain 100% of the days in the intervention. For the 8 feasibility metrics reported separately for easier versus harder goal levels, the harder level met 5 of 8 benchmarks, while the easier level met four of 8 benchmarks. According to self‐reports, feedback was reviewed weekly (i.e., as intended) by 97% of participants (29/30). No adverse events were reported.

**TABLE 3:** Feasibility outcomes compared to a priori benchmarks by goal difficulty.

Feasibility metric	All participants^a^	Among those receiving easier goal^b^	Among those receiving harder goal	Benchmark (%)
Recruitment, *n* (%)				
% of individuals enrolled among those eligible from the online screen	32 (52%)	—	—	50
Intervention engagement over 10 weeks, median [IQR]				
% days self‐monitoring dietary intake/calories	76% [38%–93%]	54% [7%–93%]	79% [41%–92%]	75
% days self‐monitoring steps	99% [95%–100%]	98% [87%–100%]	99% [96%–100%]	75
% days self‐monitoring eating window	94% [72%–99%]	93% [69%–99%]	95% [77%–99%]	75
% days self‐monitoring Red Zone Foods	93% [68%–100%]	82% [68%–100%]	95% [80%–100%]	75
% days self‐monitoring body weight	89% [63%–99%]	—^c^	—	75
% lessons reviewed	100% [89%–100%]	—	—	80
% action plans completed	100% [78%–100%]	—	—	80
Goal attainment over 10‐week intervention, median [IQR]				
% days met the daily calorie goal	28% [11%–46%]	20% [6%–54%]	29% [13%–41%]	75
% days met the daily step goal	45% [36%–51%]	51% [44%–58%]	38% [35%–45%]	75
% days met the daily eating window goal	75% [52%–93%]	78% [60%–94%]	75% [47%–93%]	75
% days met the daily Red Zone Food goal	54% [23%–79%]	65% [46%–94%]	42% [0%–63%]	75
% days met the daily protein goal	1% [0%–14%]	—	—	75
% weeks met the weekly weight loss goal	30% [20%–40%]	—	—	None set
Assessment completion, *n* (%)				
% retention (weight) at 4 weeks	31 (97%)	—	—	80
% retention (weight) at 10 weeks	30 (94%)	—	—	80
% survey completion at baseline	32 (100%)	—	—	80
% survey completion at 4 weeks	31 (97%)	—	—	80
% survey completion at 10 weeks	30 (94%)	—	—	80

*Note:* Self‐monitoring and goal attainment data are out of all days prescribed (see Methods section).

Abbreviation: IQR, interquartile range.

^a^ Feasibility outcomes (except enrollment rate) were analyzed among all 32 participants in the pilot trial.

^b^ Data reported in the easier and harder columns refer to the 4 goal domains that were experimentally manipulated (the calorie goal, step goal, eating window goal, and Red Zone Food goal). For example, data pertaining to the calorie goal and self‐monitoring dietary intake/calories are presented for those receiving the easier versus harder calorie goal.

^c^ —, indicates that this metric was not compared by goal difficulty.

### 3.5 Acceptability Outcomes

Among all participants, acceptability benchmarks were met for 9 of 14 (64%) metrics (Table 4). The harder level met four of its 6 acceptability benchmarks while the easier level met 3 of its 6 benchmarks. Among all 13 of the intervention components evaluated in terms of helpfulness, the components focused on tracking (i.e., self‐monitoring) weight, food, and steps were rated as most helpful, with ≥ 90% of participants endorsing them as *moderately*, *very*, or *extremely* helpful.

**TABLE 4:** Acceptability outcomes and proof of concept compared to a priori benchmarks by goal difficulty.

Metric	Sample, *N* (%)			Benchmark (%)
	All participants	Among those receiving easier goal^a^	Among those receiving harder	
Acceptability assessed at 10 weeks^b^				
% of participants who would recommend the weight loss program to a friend who is trying to lose weight	29 (97%)	—	—	80
% of participants who indicated that having a [goal] was *moderately*, *very*, or *extremely* helpful^c^				80
Calorie goal	22 (73%)	9 (64%)	13 (81%)	
Step goal	25 (83%)	11 (79%)	14 (88%)	
Eating window goal	24 (80%)	15 (94%)	9 (64%)	
Red Zone Food goal	21 (70%)	10 (71%)	11 (69%)	
Protein goal	22 (73%)	—	—	
10‐week weight loss goal	25 (83%)	—	—	
Weekly weight loss goal	23 (77%)	—	—	
% of participants who indicated that [intervention component] was *moderately*, *very*, or *extremely* helpful^c^				80
Tracking foods every day	27 (90%)	13 (93%)	14 (88%)	
Tracking steps every day	29 (97%)	13 (93%)	16 (100%)	
Tracking weight every day	29 (97%)	—	—	
Receiving a personalized progress report each week	24 (80%)	—	—	
Receiving a lesson each week	24 (80%)	—	—	
Filling out an action plan	22 (73%)	—	—	
Proof of concept: Clinically significant weight loss from baseline to 10 weeks				
% of participants with ≥ 3% weight loss	18 (56%)	—	—	≥ 50
% of participants with ≥ 5% weight loss	9 (28%)	—	—	≥ 33

^a^ Data reported in the easier and harder columns refer to the 4 goal domains that were experimentally manipulated (the calorie goal, step goal, eating window goal, and Red Zone Food goal). For example, data pertaining to the calorie goal and self‐monitoring dietary intake/calories are presented for those receiving the easier versus harder calorie goal.

^b^ Acceptability outcomes were reported by 30 participants who completed the 10‐week survey.

^c^ Helpfulness rating response options included the following: Not at all helpful, Somewhat helpful, Moderately helpful, Very helpful, and Extremely helpful. Helpfulness of tracking Red Zone Foods was not assessed since that component occurred daily only in weeks 1 and 10, and otherwise occurred only once per week.

### 3.6 Proof of Concept

Among all participants, the 3% weight loss benchmark was met but not the 5% weight loss benchmark (Table 4). Mean (SD) weight change from baseline to 10 weeks was −3.3 (2.5) kg (i.e., −7.3 (5.5) lbs.) in intent‐to‐treat analyses and −4.0% (3.6%).

In exploratory analyses, there was a main effect of the calorie goal on 10‐week weight change, whereby there was greater mean (SD) weight loss for participants with the harder calorie goal, −4.4 kg (3.4), compared to those with the easier calorie goal, −2.1 kg (3.6), with a between‐arm difference of −2.3 kg [95% CI: −4.1, −0.6 kg] or −5.1 lbs [−9.0, −1.3 lbs.]. No other main effects were found (Supporting Information S1: Table S2). Significant two‐way interactions were found, whereby the combination of the harder calorie goal with the harder version of either the step, eating window, or Red Zone Food goal resulted in greater weight loss than configurations where either goal was set to the easier version (Supporting Information S1: Table S2).

Descriptively, for the four experimentally manipulated goals, the following changes from baseline to 10 weeks were observed: a mean (SD) change in −369.6 (483.3) kcal/day for the easier calorie goal versus −490.5 (776.5) kcal/day for the harder calorie goal; 528 (1754) steps/day and 1.0 (23.1) units on the GLTEQ MVPA score index for the easier step goal versus 790 (2599) steps/day and 9.3 (21.0) MVPA score index units for the harder step goal; −1.3 (2.5) hours/day for the easier eating window goal versus −2.7 (3.1) hours/day for the harder eating window goal; and −2.9 (1.8) Red Zone Foods/day for the easier Red Zone Food goal versus −2.5 (1.4) Red Zone Foods/day for the harder Red Zone Food goal. For the step goal, mean (SD) step count per day during the 10‐week intervention was 8015 (3877) for the easier version versus 9525 (3092) for the harder version. Descriptively, among all participants, reductions over time were observed, on average, for body weight, caloric intake, eating window duration, and Red Zone Food intake, whereas increases were observed for step count, physical activity, and protein intake (Supporting Information S1: Table S3).

## 4 Discussion

The Ignite pilot optimization trial assessed the feasibility, acceptability, and proof of concept of a fully digital weight loss intervention, along with its varying difficulty levels for four goals, among adults with overweight or obesity. This is the first study to experimentally manipulate goal difficulty across multiple goal domains in a weight loss intervention. All benchmarks for feasibility of the study procedures were exceeded, with high rates of recruitment, retention, and survey completion. The trial was able to be conducted from start to stop within 4.5 months.

Likewise, benchmarks were met for intervention engagement. Most participants completed all weekly action plans and had high self‐monitoring across domains (dietary intake, steps, eating window, Red Zone Foods, and body weight) throughout the 10‐week intervention. This high engagement was notable given the high number of goal domains (i.e., 4–5) to track per day across four digital tools (a smart scale, an activity tracker, an app, and a e‐checklist). High engagement may be attributable to the ease of use and/or perceived usefulness of engaging, along with the integration of evidence‐based components (goal setting, self‐monitoring, feedback, and action planning) that have been shown to work in tandem to promote greater behavior change [5, 59, 60].

One notable finding was that goal attainment was low for all domains except for the daily eating window goal. This goal may have been easier to meet, relative to the others, given that it simply required knowing and sticking to one's start and stop time for eating during the day, whereas other goals required more mental or app‐based calculations (i.e., for the Red Zone Food, calorie, and protein goals) or more effort to change behavior throughout the day (i.e., the step goal). This is an important mechanism to be tested in large‐scale trials, given that goal setting theory posits that low goal attainment—such as due to goals being too difficult to achieve—leads to dissatisfaction, disengagement, a reduction in self‐efficacy, goal abandonment, and reduced performance [7]. In weight loss studies, lower goal attainment is associated with less weight loss [110, 111]. As expected, the easier version of the step and Red Zone Food goals had greater goal attainment than the harder version, descriptively, aligning with research from digital physical activity interventions [29]. Surprisingly, goal attainment was similar for the harder and easier versions of the calorie goal, which may be explained by the easier calorie goal still being considered hard to achieve by 62% of participants—on par with that of the harder calorie goal. Of note, the goal attainment benchmark of goals being met at least 75% of days may have been unrealistically high, though there has been research in the learning sciences that 85% goal achievement may be optimal for student performance [112].

High acceptability of the digital weight loss intervention was established, with nearly all participants indicating they would recommend it to a friend trying to lose weight. Further, participants considered most of the intervention components to be helpful, with the highest rated components being the daily self‐monitoring strategies (food, steps, and weight). The lowest rated components (calorie goal, Red Zone Food goal, protein goal, and action plans) were still considered helpful by ≥ 70% of participants. Notably, the protein goal had near‐zero goal attainment, indicating that it may have been out‐of‐reach for most individuals.

One of the implicit purposes of the Ignite pilot trial was to understand whether goal difficulty levels were successfully manipulated for each of the four experimental goals, with sufficient spread between the harder and easier versions. The *easier* versions of goals were rated as *somewhat* or *very* easy by most participants (85%–100%) for 3 of the 4 goal domains: steps, eating window, and Red Zone Foods. For the *harder* versions of the goals, goals were rated as *somewhat* or *very* hard by a low of only 44% of participants with the harder step goal to a high of 69% of participants with the harder eating window. These data suggest that there may be some room to test even harder versions of goals.

For proof of concept, the Ignite digital weight loss intervention resulted in an average of 3.3 kg weight loss over 10 weeks, with 56% of participants achieving 3% weight loss, and 28% achieving 5% weight loss—demonstrating a plausible clinical signal. In exploratory analyses, the pilot study revealed that the harder calorie goal produced 2.3 kg of greater weight loss than the easier calorie goal, with weight loss enhanced further when it was paired with the harder versions of any of the step, eating window, and Red Zone Food goals. This difference in weight loss may have occurred given that the harder version of the calorie goal, compared to the easier version, had greater engagement in self‐monitoring dietary intake, greater calorie goal attainment, and greater reduction in caloric intake. Past studies have shown that relatively easier goals akin to a “small change approach” intervention—which typically prescribe reductions of 100–200 kcal/day—have minimal effect on weight loss [113, 114]; in comparison, the easier calorie goal that was tested in the Ignite study produced an average reduction in caloric intake of 370 kcal/day, signaling that it still required effortful dietary change.

Study strengths include (1) use of a factorial RCT design to experimentally manipulate goal difficulty across 4 goal domains; (2) use of benchmarks for feasibility, acceptability and proof of concept that were established prior to the start of the trial; (3) use of a fully remote trial design to aid in broader reach, faster enrollment, and increased convenience to participants; (4) use of an automated digital intervention to facilitate greater scalability and lower costs; and (5) objective data collection of body weight and step count via digital tools as well as objective data on frequency of self‐monitoring and action plan completion.

This study had several limitations. First, relying on self‐report data to capture lesson completion rates, frequency of feedback review, and actual eating windows, Red Zone Food intake, and dietary intake may lower validity and introduce social desirability bias and recall bias. Second, having 34 metrics with accompanying benchmarks spanning feasibility (*n* = 18), acceptability (*n* = 14), and proof of concept (*n* = 2) made it difficult to succinctly identify whether progression to a fully powered trial is warranted. Future pilot trials should consider identifying three to five priority metrics for progression [115], with secondary metrics used for refinements. Use of “minimal viable dose” versus target dose as benchmarks for progression could also be considered, as recommended previously [116, 117]. Third, given the pilot nature of this trial, weight loss effects should be interpreted with caution, and the brief nature of the intervention is unlikely to produce clinically significant weight loss. At this stage of research, 3%–5% weight loss was selected as markers of proof of concept over 10 weeks to identify whether a large‐scale, longer clinical trial is justified to evaluate efficacy outcomes. Fourth, we did not assess step count before the intervention began, precluding our ability to establish a typical daily step count, as recommended [118].

The above considerations support two main refinements to the Ignite intervention goals prior to proceeding to a future fully powered optimization trial: (1) remove the Red Zone Food goal as an experimental domain since it overlapped with the calorie goal's purpose and was only tracked weekly in the majority of weeks; and (2) experimentally manipulate the protein goal given its near‐zero goal attainment and potential to impact body composition during weight loss efforts. A future optimization trial would differ from the present pilot optimization trial in that it would recruit a larger sample size to have sufficient power to test main effects and interaction effects and have an extended intervention length to examine longer‐term effects. An “optimization objective” was selected as identifying the combination of goal difficulty levels that maximizes weight loss while minimizing undue patient burden.

## 5 Conclusion

In sum, the Ignite pilot optimization trial demonstrated feasibility of study procedures, high engagement, and moderate‐to‐high acceptability, supporting progression to a fully powered optimization‐RCT following minor refinements. This study can serve as a model for researchers using the MOST framework to conduct of a pilot optimization trial of a fully digital intervention. Ultimately, this line of research aims to advance the science of goal setting by generating first‐ever empirical data on the optimal goal difficulty level across multiple goal domains in a weight loss intervention for adults with overweight or obesity. It will also add to the expanding literature on optimizing digital weight loss interventions [85, 101, 119, 120, 121, 122, 123] to maximize their efficacy while balancing real world constraints.

## Author Contributions

M.L.P. conceived of and designed the study. M.L.P. and L.G.R. acquired funding. L.G.R. and M.A.O. assisted with the methodology. M.L.P., A.B.Z., and P.C.C. administered the project. A.B.Z., P.C.C., K.A.C., and A.W.L. delivered the intervention and collected data. J.A.G. and M.L.P. conducted statistical analyses. M.L.P drafted the manuscript. All authors were involved in editing the paper and had final approval of the submitted and published versions.

## Funding

This study was funded by a grant from the Stanford Diabetes Research Center Pilot & Feasibility Grants Program (PI: M.L.P.) under award P30DK116074 from the National Institute of Diabetes and Digestive and Kidney Diseases (NIDDK) of the U.S. National Institutes of Health (NIH). M.L.P. received partial support from NIDDK/NIH grant K23DK129805. The content is the sole responsibility of the authors and does not necessarily represent the official views of the NIH.

## Conflicts of Interest

The authors declare no conflicts of interest.

## Supporting information


Supporting Information S1

## Data Availability

The data that support the findings of this study are available from the corresponding author upon reasonable request.
