# Supplementary material for: Optimizing Goal Difficulty in a Digital Weight Loss Intervention: The Ignite Pilot Randomized Trial
Source: Obes Sci Pract. 2026 Jul 19;12(4):e70175. doi: 10.1002/osp4.70175 (PMC13380974; doi:10.1002/osp4.70175)
Supplement: Supplementary file 1 — Supporting Information S1 [file OSP4-12-e70175-s001.pdf]

Supporting Information

Patel ML, Gallis JA, Zeitlin AB, Crosthwaite PC, Lim AW, Collins KA, Oppezzo MA, Rosas LG. Optimizing goal difficulty in a digital weight loss intervention: The Ignite pilot randomized trial. *Obesity Science & Practice*.

Michele L. Patel[1], John A. Gallis[2,3], Amanda B. Zeitlin[1,4], Phoebe C. Crosthwaite[1,5], Annalisa W. Lim[6,7], Kayla A. Collins[6], Marily A. Oppezzo[1], Lisa G. Rosas[8,9]

[1] Stanford Prevention Research Center, Department of Medicine, Stanford University School of Medicine, Palo Alto, CA, USA
[2] Duke Global Health Institute, Duke University, Durham, NC, USA
[3] Department of Biostatistics & Bioinformatics, Duke University, Durham, NC, USA
[4] Department of Pediatrics, Stanford University School of Medicine, Palo Alto, CA, USA
[5] Department of Innovation and Emerging Technology, Children's Hospital Los Angeles, Los Angeles, CA, USA
[6] Stanford University, Stanford, CA, USA
[7] Johns Hopkins Bloomberg School of Public Health, Baltimore, MD, USA
[8] Department of Epidemiology & Population Health, Stanford University School of Medicine, Palo Alto, CA, USA
[9] Division of Primary Care and Population Health, Department of Medicine, Stanford University School of Medicine, Palo Alto, CA, USA

Corresponding Author:
Michele L. Patel, PhD
Stanford Prevention Research Center
Department of Medicine, Stanford University School of Medicine
3180 Porter Drive
Palo Alto, CA 94304, USA
Email: michele.patel@stanford.edu

Table S1. Baseline characteristics of Ignite participants.

Characteristic	Total (N=32)
**Clinical characteristics**	
Weight, kg, mean (SD)	85.7 (11.9)
BMI, kg/m^2, mean (SD)	30.3 (3.6)
BMI category, N (%)	
Has overweight, 25-29.9 kg/m^2	18 (56.3)
Has obesity, 30-45.0 kg/m^2	14 (43.8)
Limited health literacy (NVS), N (%)	4 (12.5)
Prediabetes or Type 2 diabetes, N (%)	9 (28.1)
Hypertension, N (%)	6 (18.8)
**Sociodemographic characteristics**	
Age, y, mean (SD)	47.7 (13.3)
Gender, N (%)	
Man	8 (25.0)
Woman	23 (71.9)
Gender non-binary	1 (3.1)
Sex assigned at birth, N (%)	
Male	8 (25.0)
Female	24 (75.0)
Sexual orientation, N (%)	
Straight	29 (90.6)
Lesbian or gay	2 (6.3)
Queer	1 (3.1)
Marital status, N (%)	
Married or living with partner	19 (59.4)
Not married or living with partner	15 (46.9)
Race/ethnicity, N (%)	
Hispanic (any race)	5 (15.6)
Non-Hispanic White	17 (53.1)
Non-Hispanic Black	5 (15.6)
Non-Hispanic Asian/Native Hawaiian/Pacific Islander	3 (9.4)

Non-Hispanic Multi-Racial	2 (6.3)
Education, N (%)	
High school graduate	2 (6.3)
Some college	5 (15.6)
Associate degree	2 (6.3)
College graduate (4 years) or more	23 (71.9)
Employment status, N (%)[b]	
Employed full-time	20 (62.5)
Employed part-time	4 (12.5)
Student	3 (9.4)
Retired	4 (12.5)
Not employed	2 (6.3)
Annual household income, US $, N (%)	
$0-$49,999	6 (18.8)
$50,000-$99,999	10 (31.3)
$100,000 or greater	15 (46.9)
Unknown	1 (3.1)
Type of smartphone, No. (%)	
iPhone	26 (81.3)
Android	6 (18.8)
Owns a fitness tracker or smartwatch, No. (%)	24 (75.0)
**Behavioral characteristics**	
Self-monitoring of diet in the past month, N (%)	
Daily	2 (6.3)
1 to 6 times per week	4 (12.5)
< 1 time per week	6 (18.8)
Never tracked diet	20 (62.5)
Self-monitoring of steps in the past month, N (%)	
Daily	13 (40.6)
1 to 6 times per week	5 (15.6)
< 1 time per week	3 (9.4)
Never tracked steps	11 (34.4)
Self-monitoring of weight in the past month, N (%)	

Daily	8 (25.0)
1 to 6 times per week	8 (25.0)
< 1 time per week	11 (34.4)
Never tracked weight	5 (15.6)
Caloric intake, kcal/day (ASA24), mean (SD)	1950.9 (673.1)
Step count, steps/day[c] (Fitbit Inspire 3), mean (SD)	8787 (3773)
Physical activity (GLTEQ)	
MVPA score index, mean (SD)	25.0 (22.8))
Leisure score index, mean (SD)	35.2 (28.9)
Active, N (%)	19 (59.4)
Moderately active, N (%)	6 (18.8)
Insufficiently active, N (%)	7 (21.9)
Physical activity guideline adherence (L-Cat)	
Met guidelines	7 (21.9)
Did not meet guidelines	25 (78.1)
Eating window	
Eating window duration, hours/day, mean (SD)	11.7 (2.9)
Eat ≥12 hours/day, N (%)	18 (56.3)
Eating start time, hh:mm, mean (SD)	09:06 AM (02:10)
Eating end time, hh:mm, mean (SD)	08:50 PM (02:25)
Red Zone Foods intake, count/day (ASA24), mean (SD)	5.9 (2.4)
Protein intake, grams/day (ASA24), mean (SD)	78.1 (28.6)

Abbreviations: ASA24, Automated Self-Administered 24-Hour (ASA24) Dietary Assessment Tool; BMI, body mass index; GLTEQ, Godin Leisure-Time Exercise Questionnaire; hh:mm, hours (2 digits) and minutes (2 digits) of time-of-day using the 12-hour clock; kcal, kilocalorie; L-Cat, Stanford Leisure-Time Activity Categorical Item; MVPA, moderate-to-vigorous physical activity; NVS, Newest Vital Sign health literacy measure.

[a] No participants who identified as Non-Hispanic selected 'American Indian or Alaska Native'.

[b] Numbers add up to >100% because participants could select multiple employment options.

[c] Reported data refer to the mean step count in week 1 since participants did not begin wearing the Fitbit tracker until the start of the intervention.

Table S2. Main effects and interaction effects of intervention components on weight change.

Effect	Weight change from baseline, kg (95% CI)	
	4 Weeks	10 Weeks
**Main effects of intervention components: harder goal (+1) vs. easier goal (-1)**		
Calorie goal	**-1.6 (-3.1, -0.2)**	**-2.3 (-4.1, -0.6)**
Step goal	-0.7 (-2.2, 0.7)	-0.7 (-2.5, 1.0)
Eating window goal	-1.1 (-2.6, 0.4)	0.1 (-1.7, 1.8)
Red Zone Foods goal	-0.3 (-1.8, 1.1)	-0.0 (-1.8, 1.7)
**2-way interaction effects of intervention components**		
Calorie x step	**-1.6 (-3.0, -0.1)**	**-2.0 (-3.7, -0.2)**
Calorie x eating window	-0.8 (-2.3, 0.7)	**-2.5 (-4.3, -0.8)**
Calorie x Red Zone Foods	-0.8 (-2.2, 0.7)	**-1.7 (-3.5, 0.02)**
Step x eating window	-1.1 (-2.6, 0.4)	**-2.1 (-3.8, -0.3)**
Step x Red Zone Foods	-0.4 (-1.9, 1.1)	-1.0 (-2.7, 0.8)
Eating window x Red Zone Foods	-0.5 (-2.0, 1.0)	-1.4 (-3.2, 0.3)
**3-way interaction effects of intervention components**		
Calorie x step x eating window	-0.5 (-2.0, 1.0)	0.2 (-1.6, 1.9)
Calorie x step x Red Zone Foods	-1.1 (-2.6, 0.4)	-1.4 (-3.1, 0.4)
Calorie x eating window x Red Zone Foods	-0.0 (-1.5, 1.4)	0.2 (-1.6, 1.9)
Step x eating window x Red Zone Foods	**-1.9 (-3.3, -0.4)**	-1.5 (-3.2, 0.3)
**4-way interaction effects of intervention components**		
Calorie x step x eating window x Red Zone Foods	-0.8 (-2.3, 0.7)	**-1.9 (-3.6, -0.2)**

Abbreviations: Calorie, calorie goal; Eating window, eating window goal; Red Zone Foods, Red Zone Foods goal; Step, step goal.

Notes: Bolded, green values indicate $p < .05$. Harder goals were coded as +1 while easier goals were coded as -1. To interpret the main effects, a main effect of the calorie goal of 2.3 kg represents the *harder* calorie goal having 2.3 kg greater weight loss at 10 weeks than the *easier* calorie goal. Data are reported to show proof of concept and are not powered to detect differences in efficacy. *P* values are not reported given that this study was not powered to detect statistically significant effects.

Table S3. Exploratory weight and behavior outcomes, by goal domain and goal difficulty.[a]

Outcome	All participants (N=32)	Goal domain and goal difficulty level							
		Calorie goal		Step goal		Eating window goal		Red Zone Food goal	
		Easier (n=16)	Harder (n=16)	Easier (n=16)	Harder (n=16)	Easier (n=16)	Harder (n=16)	Easier (n=16)	Harder (n=16)
Weight, kg, mean (SD)									
0-4 weeks, Δ	-1.4 (2.1)	-0.6 (3.1)	-2.2 (2.9)	-1.1 (3.1)	-1.8 (2.9)	-0.9 (2.9)	-2.0 (3.1)	-1.3 (3.1)	-1.6 (2.9)
0-10 weeks, Δ	-3.3 (2.5)	-2.1 (3.6)	-4.4 (3.4)	-2.9 (3.6)	-3.6 (3.4)	-3.3 (3.4)	-3.2 (3.6)	-3.3 (3.6)	-3.3 (3.4)
Relative weight, %, mean (SD)									
0-4 weeks, Δ	-1.7 (2.4)	-1.0 (2.4)	-2.4 (2.3)	-1.3 (2.3)	-2.0 (2.6)	-1.3 (2.4)	-2.0 (2.5)	-1.8 (2.0)	-1.6 (2.8)
0-10 weeks, Δ	-4.0 (3.6)	−3.1 (3.7)	−4.9 (3.3)	-3.7 (3.8)	-4.4 (3.4)	-4.5 (2.6)	-3.6 (4.3)	-4.3 (2.8)	-3.8 (4.2)
Proportion with clinically significant weight loss at 10 weeks, N (%)									
≥3% weight loss	18 (56%)	5 (31%)	13 (81%)	9 (56%)	9 (56%)	10 (62.5)	8 (50.0)	10 (62.5)	8 (50.0)
≥5% weight loss	9 (28%)	4 (25%)	5 (31%)	5 (31%)	4 (25%)	6 (37.5)	3 (18.8)	5 (31.3)	4 (25.0)
Caloric intake, kcal/day[b] (ASA24), mean (SD)									
0-10 weeks, Δ	-397.6 (632.7)	-382.0 (466.6)	-412.3 (773.3)	-490.0 (517.9)	-322.6 (720.7)	-344.7 (596.7)	-454.4 (687.1)	-478.7 (664.5)	-321.9 (614.8)
Step count, steps/day, (Fitbit Inspire 3 tracker), mean (SD)[c]									
0-10 weeks, Δ	673 (2226)	--	--	528 (1754)	790 (2599)	--	--	--	--
Per day during intervention	8794 (3519)			8015 (3877)	9525 (3092)				
Physical activity (GLTEQ)									
Leisure score index, mean (SD)									
0-4 weeks, Δ	5.0 (21.9)	--	--	-0.6 (22.6)	10.3 (20.5)	--	--	--	--
0-10 weeks, Δ	9.7 (21.7)	--	--	4.6 (23.0)	14.1 (20.3)	--	--	--	--
MVPA score index, mean (SD)									
0-4 weeks, Δ	1.4 (19.0)	--	--	-2.6 (17.6)	5.1 (20.1)	--	--	--	--
0-10 weeks, Δ	5.4 (22.0)	--	--	1.0 (23.1)	9.3 (21.0)	--	--	--	--
Proportion of participants meeting physical activity guidelines (L-Cat) over time, N (%)[d]									
Met guidelines initially: continued to do so at 10 weeks	6 (20.0)	--	--	4 (28.6)	2 (12.5)	--	--	--	--
Met guidelines initially: no longer did at 10 weeks	0 (0.0)	--	--	0 (0.0)	0 (0.0)	--	--	--	--
Did not meet guidelines initially: increased activity level at 10 weeks	15 (50.0)	--	--	5 (35.7)	10 (62.6)	--	--	--	--
Did not meet guidelines initially: decreased or maintained activity level at 10 weeks	9 (30.0)	--	--	5 (35.7)	4 (25.0)	--	--	--	--
Eating window duration, hours/day, mean (SD)									
0-10 weeks, Δ	-1.9 (2.8)	--	--	--	--	-1.3 (2.5)	-2.7 (3.1)	--	--
Red Zone Foods intake, count/day[b] (ASA24), mean (SD)									
0-10 weeks, Δ	-2.7 (1.6)	-2.3 (1.8)	-3.0 (1.3)	--	--	--	--	-2.9 (1.8)	-2.5 (1.4)
Protein intake, grams/day[b] (ASA24), mean (SD)									
0-10 weeks, Δ	3.1 (29.5)	-0.5 (32.6)	6.5 (26.9)	--	--	--	--	1.6 (33.3)	4.5 (26.5)

Abbreviations: 0-10 weeks, baseline assessment to 10-week assessment; ASA24, Automated Self-Administered 24-Hour Dietary Assessment Tool; GLTEQ, Godin Leisure-Time Exercise Questionnaire; kcal, kilocalorie; MVPA, moderate-to-vigorous physical activity.

Note. This table depicts the planned outcomes of a subsequent, fully powered optimization-RCT. All results reflect the raw values changes from baseline, except for change in daily step count, which refers to changes from week 1's mean step count (since participants did not begin wearing the Fitbit tracker until the start of the intervention).

[a] Body weight data in kg are presented using linear mixed models in an intent-to-treat approach; otherwise, a completer's analysis is presented for these pilot study data. Unless otherwise noted, both weight and survey data are collected from n=32/32 participants at baseline; n=31/32 participants at 4 weeks; and 30/32 participants at 10 weeks.

[b] The ASA24 dietary recall data are reported for all 32 participants at baseline and 29 of 32 participants at 10 weeks due to missing or invalid data.

[c] Step count was collected via the Fitbit Inspire 3 activity tracker; data are collected from n=31/32 in week 1 and 27/32 in week 10.

[d] Step count Physical activity guideline adherence data measured from the L-CAT are reported for the 30/32 participants who completed the 10-week survey.
